# A national atlas of tsetse flies and African animal trypanosomosis in Ghana

**DOI:** 10.1186/s13071-026-07725-2

**Published:** 2026-10-03

**Authors:** Benita Anderson, Yahaya Adam, Charles Mahama, Yakubu Sakara, Enoch Sottie, Massimo Paone, Giuliano Cecchi

**Affiliations:** 1 Tsetse and Trypanosomosis Control Unit, Veterinary Services Directorate, Ministry of Food and Agriculture, Pong Tamale, Ghana; 2 Animal Health and Production College, Ministry of Food and Agriculture, Pong-Tamale, Ghana; 3 Veterinary Services Directorate, Ministry of Food and Agriculture, Ledzokuku Krowor Municipal Assembly, Ghana; 4 Tsetse and Trypanosomosis Control Unit, Veterinary Services Directorate, Ministry of Food and Agriculture, Accra, Ghana; 5 Food and Agriculture Organization of the United Nations, Animal Production and Health Division, Rome, Italy

**Keywords:** Tsetse, *Glossina*, African animal trypanosomosis, GIS, Atlas, Ghana, Epidemiology

## Abstract

**Background:**

Tsetse-transmitted African animal trypanosomosis (AAT) remains a widespread and major constraint to sustainable development and food security in vast areas of sub-Saharan Africa. In Ghana, like in most endemic countries, data on the occurrence of the tsetse vector and the disease are available at the country level, but for a long time information has been scattered and poorly harmonized, thus hampering proper analysis and evidence-based decision-making.

**Methods:**

The Tsetse and Trypanosomosis Control Unit (TTCU) in Ghana collated, harmonized, georeferenced and centralized in a single database (the ‘atlas’) all the available field data on tsetse and AAT occurrence for period 1992–2025. For the tsetse component, 3087 trapping records corresponding to 2864 trapping locations and 26,121 flies caught were included. For the AAT component, 740 herd-level records were assembled (393 survey sites and 26,653 tested animals).

**Results:**

Both tsetse and AAT were found to be widespread in Ghana. Three of the 10 historically reported tsetse species were detected: *Glossina tachinoides*, *G. palpalis*, and *G. morsitans*. *G. palpalis* is the only one with a country-wide distribution, while *G. tachinoides* is more common in the northern part of the country and *G. morsitans* populations are smaller, more fragmented and mainly confined to protected areas. In the Upper West region, major tsetse control operations were carried out in 2010, whose gains in terms of reduction of fly densities seem to have been sustained over time. As regards animal trypanosomosis, almost 90% of the data were collected on cattle. The average prevalence of bovine trypanosomosis is 7.7% (825/10,766) with the buffy coat technique, which was used on over two thirds of the randomly selected samples. Predictably, higher prevalence was measured with more sensitive but less frequently used diagnostics, i.e. antibody enzyme-linked immunosorbent assay (Ab-ELISA) (22%, 720/3277) and polymerase chain reaction (PCR) (17%, 332/1949).

**Conclusions:**

The national atlas of tsetse and AAT in Ghana, already developed by 2018, was regularly updated ever since. It is now the pillar of data management for the TTCU, and it is used to measure and drive country progress along the progressive control pathway for AAT. The open data policy will also enable to maximize the atlas reuse and impact.

**Graphical Abstract:**

**Figure:**
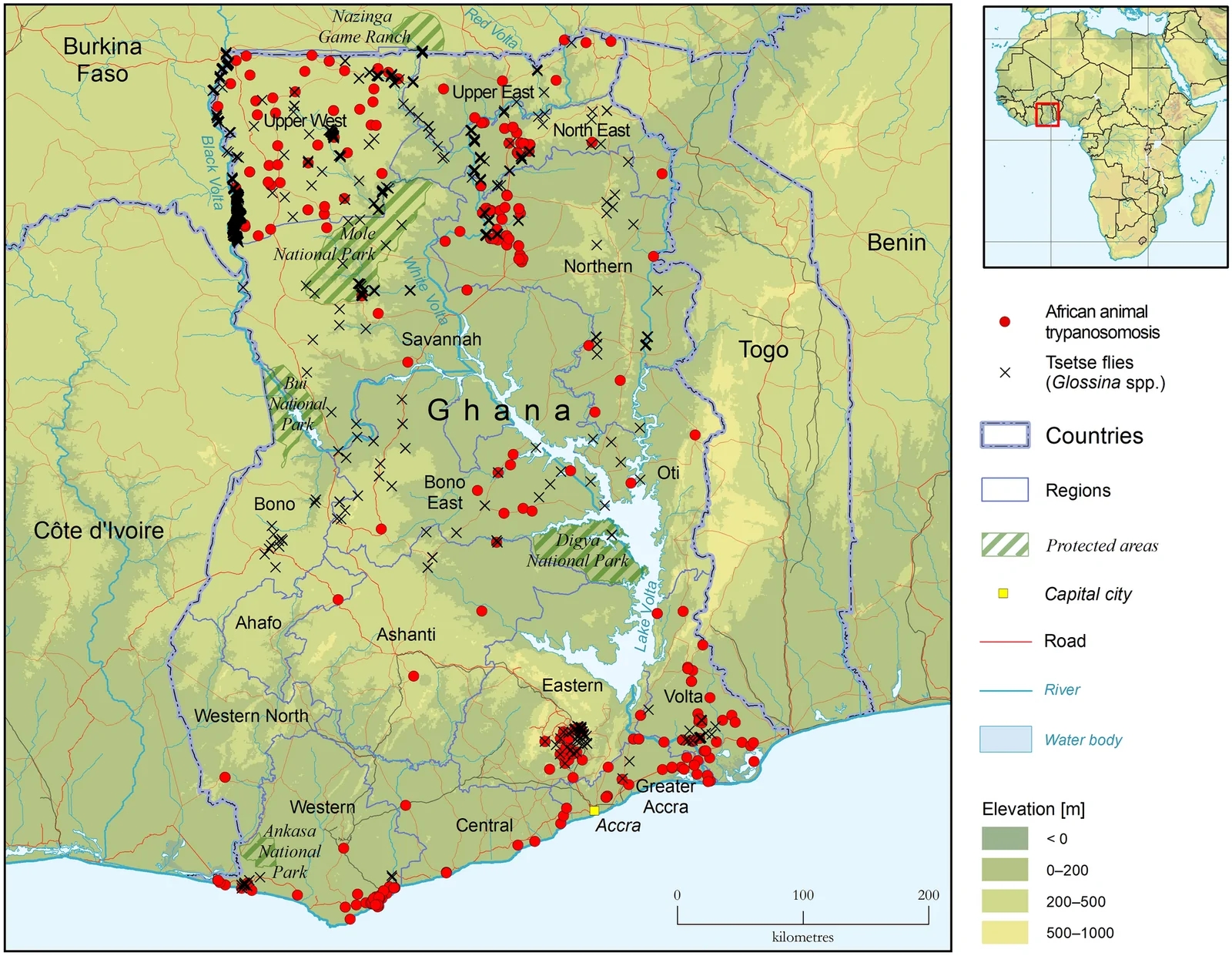

**Supplementary Information:**

The online version contains supplementary material available at https://doi.org/10.1186/s13071-026-07725-2.

## Background

African animal trypanosomosis (AAT), also known as nagana, is caused by protozoa of the genus *Trypanosoma*, and in particular by *T. vivax*, *T. congolense*, *T. brucei* and *T. simiae* [1–3]. In sub-Saharan Africa, the disease is mainly transmitted cyclically by tsetse flies (*Glossina* spp.), and it is virtually ubiquitous in the 10 million km^2^ that are believed to be infested by these flies [4]. However, AAT can also be transmitted mechanically by other biting flies (e.g. *Tabanus* and *Stomoxys* spp.) [5], and mechanical vectors are responsible for the transmission of *T. vivax* in Latin America [6], now reported also in Asia [7].

The disease causes major losses in livestock and crop agriculture, with severe socio-economic consequences [8, 9]. In Ghana, the potential for sustainable intensification of livestock–crop production is highest in areas along the main rivers, with abundant vegetation and pastures. Unfortunately, these areas are also endemic for trypanosomosis. As in most of the continent, the economic impacts of AAT in Ghana are felt most acutely in cattle [10–12], even though pig production is also affected, especially in the southern part of the country [13].

In addition to affecting animals, two subspecies of *T. brucei* (i.e. *T. b. gambiense* and *T. b. rhodesiense*) also infect humans [14]. The human form of the disease, also known as sleeping sickness or human African trypanosomiasis (HAT), has a patchy, focal distribution, and it threatens poor rural communities in approximately 700 000 km^2^ of sub-Saharan Africa [15]. However, HAT no longer represents a public health problem in Ghana [16], and the latest autochthonous case was detected in 2013, after over 10 years without any reported infection [17].

During colonial times, as early as the 1920s, tsetse and trypanosomosis received attention in Ghana because of high mortalities and morbidity along trade routes in the northern territories of what was known at the time as the Gold Coast [18]. Since then, efforts have been made to control AAT in the country. Before independence, interventions against tsetse mainly involved the drastic alteration of the tsetse habitat by selective removal of vegetation, although tsetse traps were starting to be used [18]. In the 1990s, large-scale community-based vector control operations were implemented in different parts of the country with the use of biconical traps, insecticide treated targets (ITT), insecticide treated cattle (ITC) and ground spraying, combined with chemotherapy for disease control [19]. More recently, large-scale aerial spraying of insecticide was used in the Upper West region within the framework of the Pan-African Tsetse and Trypanosomosis Eradication Campaign (PATTEC). In this area, an integrated strategy of intervention was adopted, whereby the sequential aerosol technique (SAT) was complemented by ground spraying, ITT and ITC [20].

As regards the occurrence of tsetse flies, studies carried out until the turn of the twentieth century indicated that ten species were found in Ghana (i.e. *Glossina caliginea*, *G. pallicera*, *G. palpalis* and *G. tachinoides* of the *palpalis* group; *G. morsitans* and *G. longipalpis* of the *morsitans* group; and *G. fusca*, *G. nigrofusca*, *G. tabaniformis* and *G. medicorum* of the *fusca* group [19, 21–24]). Regarding subspecies, both *G. palpalis gambiensis* and *G. palpalis palpalis* are present in Ghana [25], the former in the North and the latter in the South, while *G. morsitans submorsitans* is the only subspecies of *G. morsitans* present in the whole of western Africa, including Ghana. *Glossina fusca fusca* is the subspecies of *G. fusca* considered to be present in Ghana, while the possible presence of *G. fusca congolensis* has only been hypothesized [26]. Despite this high number of tsetse species and subspecies, a recent Africa-wide review of the peer-reviewed literature published between 1990 and 2020 only found data for four species/subspecies in Ghana: *G. morsitans submorsitans*,* G. palpalis gambiensis*, *G. palpalis palpalis* and *G. tachinoides* [4].

Between 2013 and 2018, Ghana and five other countries (i.e. Burkina Faso, Ethiopia, Kenya, Mali and Uganda) joined a FAO project to develop capacities for tsetse and trypanosomosis control [27, 28]. The project targeted the countries that were implementing the PATTEC initiative [29], and one of its main objectives was to set up information systems, also called ‘atlases’, on the geographic distribution of AAT and tsetse at the national and continental level. The continental atlas was developed by FAO by using the published literature as the sole input [4, 30, 31], while national atlases were developed by the mandated veterinary authorities, aiming to include all data that could be found at the national level, be them published or unpublished [32–35]. A national atlas was also successfully developed in Ghana by the Tsetse and Trypanosomosis Control Unit (TTCU) of the Veterinary Service Directorate (VSD), but its results were not published at the time. The present paper fills this dissemination gap, while also showing that the atlas in Ghana was continuously updated by the TTCU after the end of the FAO project. The atlas allows Ghana to assess it status and better inform decisions in the framework of the AAT progressive control pathway (PCP), a data-driven, step-wise strategic approach to reduce the burden of the disease [1, 36].

## Methods

The methodology used to develop the national atlas on tsetse and AAT in Ghana is broadly based on the FAO continental atlas [4, 30, 31]. It also benefitted from the examples provided by other national atlases [32–35, 37–40]. The atlas comprises two main elements: a data repository and a database; the former includes all input files and datasets while the latter stores the harmonized and georeferenced data that were extracted from the source files. As regards the temporal coverage, the atlas of Ghana spans over 34 years, from 1992 to 2025, and it includes both published and unpublished data.

The TTCU was the main source of data, because it is the specialized national structure in charge of tsetse and AAT surveillance and control, including outbreak investigation and response. Some TTCU data included in the atlas had been previously published in the framework of specific research activities [11, 12, 20, 41, 42]; however, the majority of the TTCU data is published here for the first time. Beyond the TTCU, additional information is collected in the field by district and regional veterinary authorities in the context of general animal disease surveillance. This information is transmitted to the veterinary authorities at the central level, namely the Epidemiology Unit of the VSD in the capital Accra, which shares the AAT-specific data with the TTCU. Finally, data from scientific publications were included, but since it was not possible to obtain the raw data from authors, FAO provided the data extracted from the publications for the development of the continental atlas.

All input data for the atlas were assembled and stored in a centralized data repository. Subsequently, the data were verified, harmonized and recorded in a geo-spatial database. Verifications were particularly needed for the geographical coordinates of the survey sites; these verifications relied on GEOnet gazetteer but also on direct communication with field staff. Furthermore, potential outliers were systematically searched and corrected using various techniques (e.g. selectively filtering or labelling data by administrative units to detect locations mapped in the wrong units). Freeware open-source software was used for data processing (i.e. QGIS), while ArcMap 10.6 was used by FAO for the preparation of the maps in this publication. Another issue that required attention was the identification and removal of duplicated records, which can stem from the use of multiple and sometimes overlapping data sources. Another challenge was obtaining raw data from a few scientific publications, which was addressed by using the FAO continental atlas as a source of harmonized data from a few scientific papers. Another issue that was verified was whether AAT records derived from surveys in which animals were randomly selected or from tests carried out on clinically suspicious animals, which is often the case in the framework of the national animal health surveillance system. This distinction between randomly and non-randomly selected animals is crucial to determine which AAT data from the atlas can be used to estimate the disease prevalence in the general livestock population. The correct handling of mixed infections was also promoted by ensuring that, following the FAO atlas approach, mixed infections are also counted in the tally of infections with each individual parasite species, so that the corresponding columns provide a complete count of infections for the different parasite species. Lastly, the names of the administrative regions were fully harmonized so that they reflect the current units, and not those of the time of data collection.

The database of the atlas is structured into three simple tables devoted to data sources, entomological data (i.e. tsetse) and epidemiological data (i.e. AAT). The tsetse distribution table includes information on the name of the trapping site, its geographical coordinates (latitude and longitude in decimal degrees) and the corresponding administrative units (region, district), the survey period, the trap type, the odour attractant (if any), the duration of trapping, as well as information on tsetse by species, i.e. absence/presence, number of flies captured and apparent density (flies/trap/day). Information on the presence of tsetse control interventions in the surveyed area, if any, is also recorded. Tsetse trapping was always carried out using biconical traps [43], with the exception of one location in the Mole National Park where flies were detected by means of moving vehicle and a hand-held net. Traps were normally deployed for one day, which was the case for approximately 80% of the entomological records, normally without olfactory attractants, which were used only in 0.6% of the cases. The trap position was georeferenced using GPS. In a separate table, data on tsetse infection are recorded. However, very little data could be identified for the component on tsetse infections, and results are not presented in this paper.

The AAT table includes the name of the survey site, its geographical coordinates and the two corresponding administrative units, survey period, diagnostic method, sample size (i.e. number of animals tested), animal species, breed, age range, sex and husbandry system. Trypanosomal infections are captured in terms of presence/absence, number of infected animals and prevalence. Mixed infections are also recorded. Lastly, information on recent or ongoing interventions against tsetse and on the use of trypanocidal drugs is included. The AAT component of the atlas relies on a combination of cross-sectional surveys based on randomly selected animals and epidemiological reports based on the testing of clinically suspicious animals. Prevalence data provided in the results section are solely based on surveys based on randomly selected animals.

## Results

### Data quantity and coverage

A total of 221 source files were included in the data repository, corresponding to 187 reports or datasets generated by the TTCU and by decentralized veterinary authorities, 24 scientific publications, 2 PhD theses and 1 article published in conference proceedings.

As regards tsetse distribution, data were compiled for 2864 different trapping locations, with the earliest dating back to 1995. This dataset corresponds to a total trapping intensity of 5104 trap days and 26,121 tsetse flies caught. Geographically, data coverage is uneven, and it is better in northern regions. As an example, almost a third of the data (i.e. 31% of the trap days) were collected in the Upper West region alone, which accounts for less than 8% of the country’s surface. An additional 31% of the data originated from the other four regions in the northern part of the country (i.e. Upper East, Northern East, Northern and Savannah). Approximately 38% of the data were collected in 8 out of the 11 regions in the southern part of the country, despite these regions accounting for almost 60% of the country’s surface. Coverage is relatively more balanced in time, with a majority of data collected in more recent period (i.e. 19%, 48% and 33%, respectively, for 1995–2004, 2005–2014 and 2015–2025).

As regards the AAT component, data for a total of 26,653 tested animals were included for the period 1992–2025, with the vast majority of them being cattle (87.9%), and the remaining being pigs (7.3%), sheep (2.4%), goats (1.9%) and dogs (0.5%). As regards cattle breeds, tested animals included both trypanotolerant (i.e. N’dama) and trypanosusceptible breeds (i.e. Zebu), with also many crossbreeds (e.g. Sanga and West Africa Short Horn). Virtually all tested animals were part of communally grazed sedentary herds.

The AAT surveys were carried out in 382 different locations. Geographically, their distribution is more balanced than the entomological surveys for tsetse, with 15,088 animals tested in the 5 northern regions and 11,565 tested in 10 out of the 11 southern regions. Temporally, fewer data were collected in more recent years, with 10,976, 9,565, 6,112 animals tested, respectively, in 1992–2002, 2003–2013 and 2014–2025. As to the diagnostic tools, traditional parasitological techniques were the most used, especially in routine surveillance activities. In particular, blood smears [44] and the buffy coat technique [45] accounted for 13.7% (3,639 animals) and 43.2% (11,518 animals) of the total tests performed. Serological techniques accounted for 15.9% of the tests, with antibody enzyme-linked immunosorbent assay (Ab-ELISA) [46] accounting for 12.3% of the tests [11, 12] and antigen-ELISA [47] accounting for 3.6% [48]. Finally, molecular techniques accounted for 13.1% of the tests (3,479 animals tested).

### Occurrence of tsetse flies and African animal trypanosomosis

**Fig. 1:**
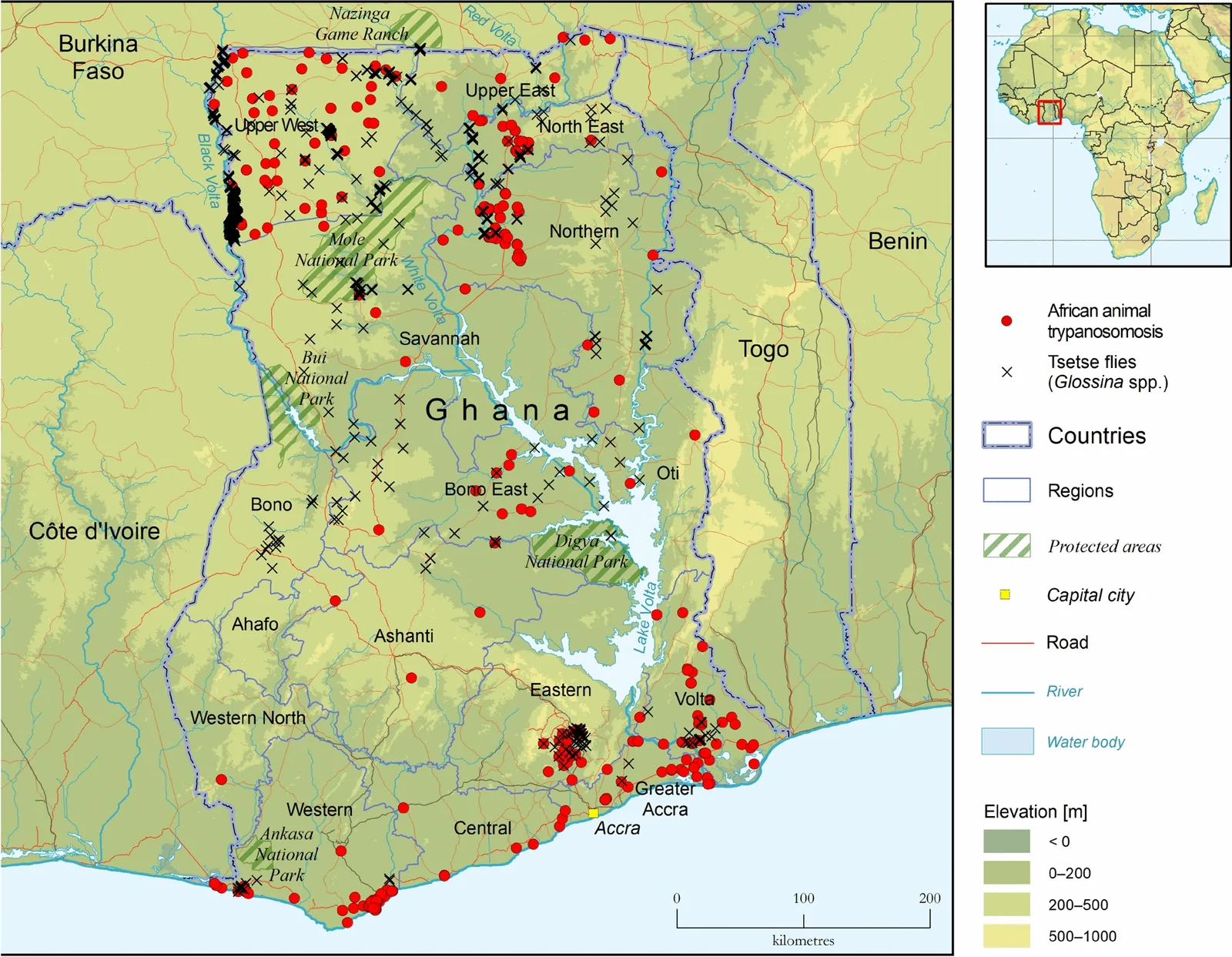
The reported occurrence of African animal trypanosomosis and tsetse flies (Genus: *Glossina*) in Ghana. Data collection period: 1992–2025

Figure 1 summarizes the national atlas of tsetse and AAT in Ghana with the reported occurrence of the vector and the disease. Points of absence of detection are not shown here, and tsetse data are mapped at the trap level wherever trap-level data are available, and at site level in other cases. Figure 1 illustrates how tsetse and AAT are widespread across Ghana, having been detected from the four corners of the country.

#### Tsetse flies

**Fig. 2:**
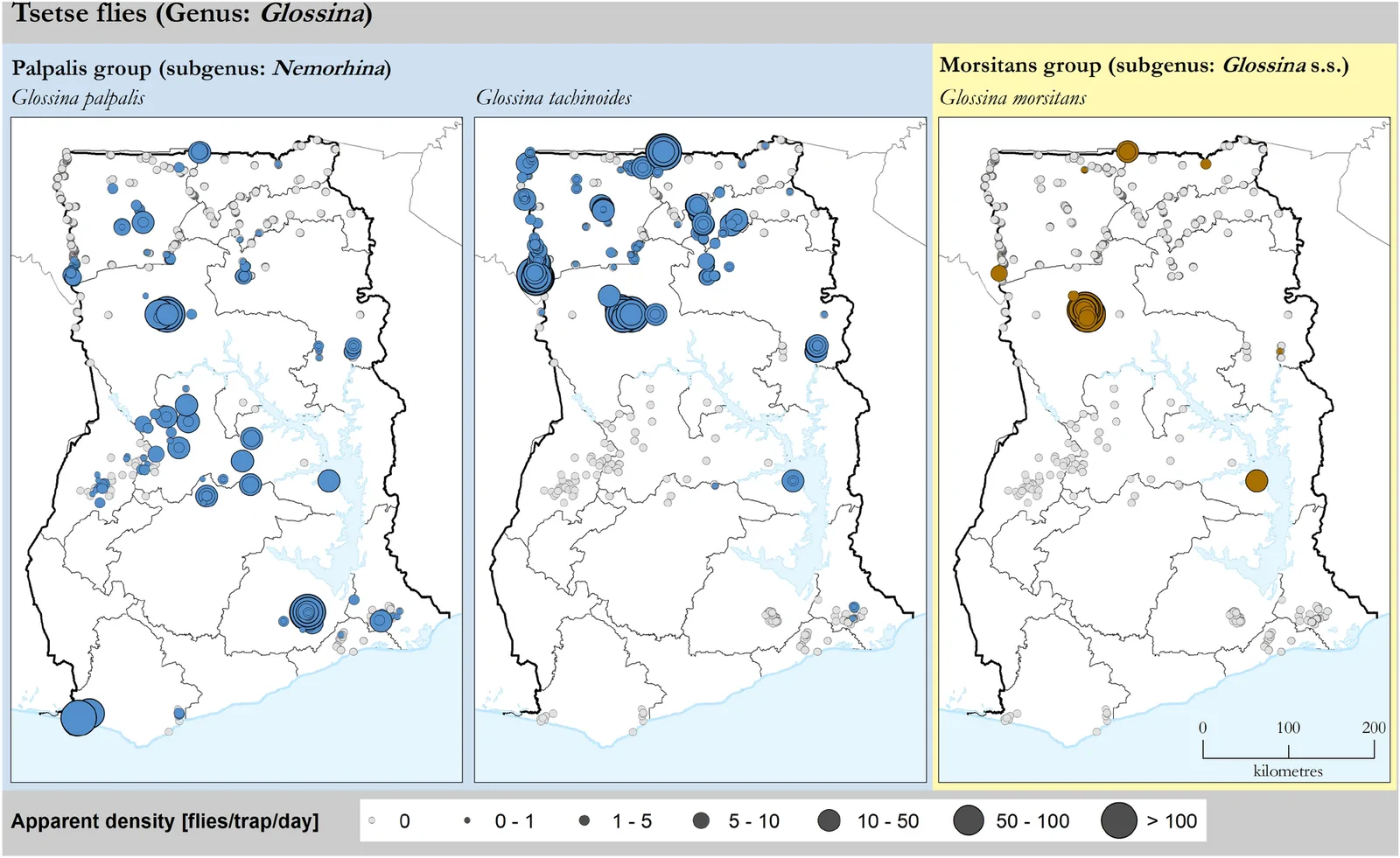
Apparent density [flies/trap/day] of *G. palpalis*, *G. tachinoides* (*palpalis *group) and *G. morsitans* (*morsitans* group) in Ghana mapped at the site level. Data collection period: 1995–2025. Trapping device: biconical trap [43]

Figure 2 and Table 1 summarize the results of the tsetse atlas at the species level. In Fig. 2 apparent density (i.e. the number of flies captured per trap and per day) is used to symbolize the results, and the points of absence of detection are also included (i.e. 0 flies/trap/day).

**Table 1:** Intensity of entomological surveillance and tsetse flies caught in Ghana. Data collection period: 1995–2024. Trapping device: biconical trap [43]

Region^1^	Intensity of surveillance			Tsetse flies caught							
	[trap days]	[No. locations]	[Period]	*G. tachinoides*		*G. palpalis*		*G. morsitans*		*Glossina* (Total)^2^	
				[No.]	AD^3^	[No.]	AD	[No.]	AD	[No.]	AD
Ashanti	110	9	1998–2005	2	0.02	68	0.62	0	0.00	72	0.65
Bono	95	45	1998	0	0.00	49	0.52	0	0.00	49	0.52
Bono East	128	33	1990–2000	38	0.30	519	4.05	122	0.95	695	5.43
Eastern	1148	78	2007–2019	0	0.00	2466	2.15	0	0.00	2466	2.15
Greater Accra	116	17	1995–2017	0	0.00	1	0.01	0	0.00	3	0.03
North East	395	391	1990–2023	591	1.50	1	0.00	0	0.00	592	1.50
Northern	219	216	1990–2022	356	1.63	135	0.62	1	0.00	492	2.25
Oti	–	4	1990–2000	–	–	–	–	–	–	–	–
Savannah	627	217	1990–2025	8032	12.81	795	1.27	1814	2.89	10,641	16.97
Upper East	353	343	1990–2023	1396	3.95	64	0.18	29	0.08	1489	4.22
Upper West	1562	1388	1990–2025	6381	4.09	407	0.26	15	0.01	6858	4.39
Volta	233	94	1998–2018	14	0.06	27	0.12	0	0.00	786	3.38
Western	119	29	2012–2016	0	0.00	1978	16.62	0	0.00	1978	16.62
Total	5104	2860	1990–2025	16,810	3.29	6510	1.28	1981	0.39	26,121	5.12

^1^No data available for Ahafo, Central and Western North regions. In Oti, tsetse presence was confirmed, but the number and the species of flies was not available

^2^*Glossina* (Total) includes 820 tsetse flies not identified at the species level

^3^Apparent density [flies/trap/day]

Figure 2 and Table 1 show that the presence of tsetse flies was confirmed in all the administrative regions investigated (i.e. 13 out of 16, with no data available for Ahafo, Central and Western North regions). In terms of geographical coverage, data were available for 56 of the 261 districts in the country (i.e. 21%), of which 43 were found to be infested by tsetse (i.e. 77% of the surveyed districts). However, in most districts where tsetse flies were not detected, the intensity of surveillance was very limited (i.e. <= 20 trap days in 16 of the 17 districts), and therefore these areas cannot be considered free of tsetse. As regards the entomological results, only three species of *Glossina* were detected: *G. tachinoides* (64% of the total trap catches), *G. palpalis* (25%) and *G. morsitans* (8%). Approximately 3% of the tsetse flies were not identified at the species level.

*G. tachinoides* (riverine group) was the most abundant and widespread species in the northern part of the country, where the intensity of surveillance was higher. This largely explains its predominance in the overall fly count at the national level. Particularly high apparent densities, which exceeded 100 flies/trap/day in a few traps, were recorded in three sites: i.e. the Black Volta River (Wa West district, Upper West region), the Mole National Park (West Gonja district, Savannah region), and the Sissala East district (Upper West region), near the Nazinga game ranch—a protected area located across the border with Burkina Faso. *G. tachinoides* was also detected in two locations in the central part of the country (i.e. Ashanti and Bono East regions) and further south in the Volta region, at less than 30 km of the Atlantic coast.

*G. palpalis*, which like *G. tachinoides* belongs to the riverine group, was detected in the North at lower densities and with a patchier distribution than *G. tachinoides*, but it was more widely distributed elsewhere. In fact, in most sites in the central and southern part of the country *G. palpalis* was the only species detected. High apparent densities for *G. palpalis*, exceeding 100 flies/trap/day in a few traps, were recorded in the Eastern region (New Juaben district), Western region (Jomoro district) and the Mole National Park.

As regards *G. morsitans* (savannah group), its distribution in Ghana is fragmented, and mostly restricted to protected areas and their surroundings (e.g. Mole and Digya national parks, Nazinga game ranch, Red Volta Forest Reserve). High densities of *G. morsitans* of more than 100 flies/trap/day were only observed in a few trapping sites within the Mole National Park. In fact, this park accounted for 75% of all the *G. morsitans* trapped in Ghana during the study period*.* Notwithstanding the key role played by protected areas, the species was also detected in three sites not directly linked to parks or other conservancies (two in the Northern region and one in the Upper Est along the Red Volta river). *G. morsitans* was also the only species detected in the Mole National Park with a moving vehicle and a hand-held net.

**Fig. 3:**
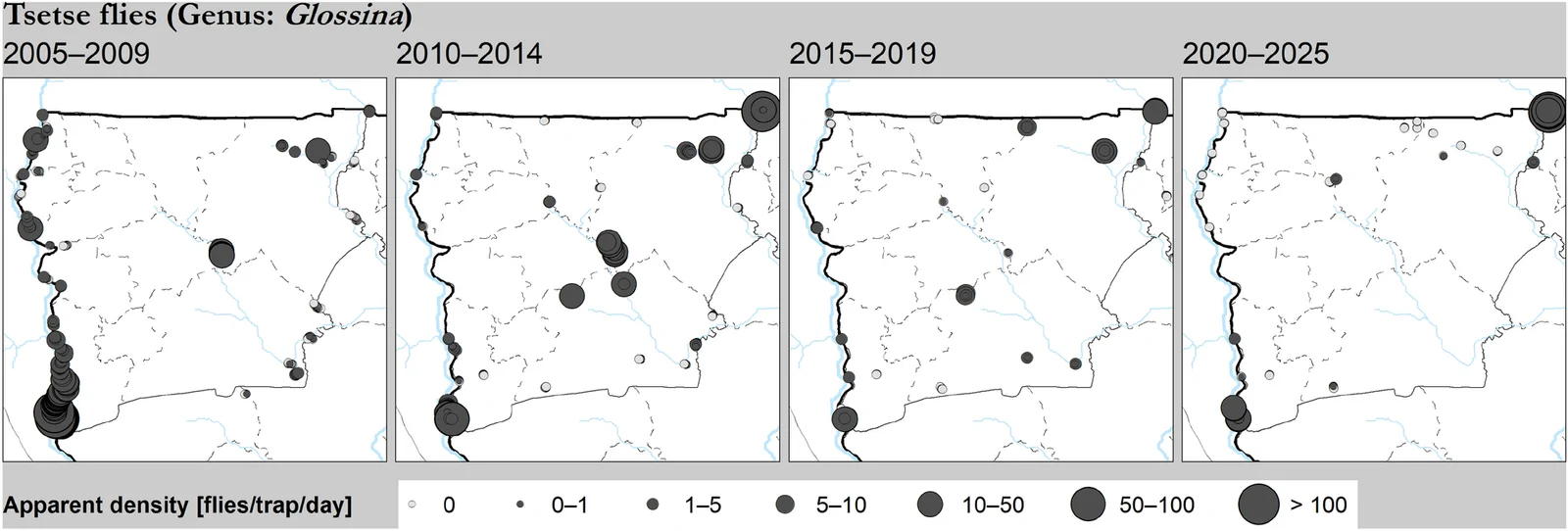
Apparent density [flies/trap/day] of *Glossina* in the Upper West region of Ghana mapped at the trap level. Periods: 2005–2009, 2010–2014, 2015–2019 and 2020–2025. Trapping device: biconical trap [43]

Figure 3 and Table 2 provide an example of how the atlas can be used to generate time series to illustrate temporal trends. The time series focuses on the Upper West region, which in the early 2010s was the target of major tsetse control operations integrating different tools. In particular, in 2010 SAT was deployed, resulting in a fast and substantial reduction of tsetse densities, although it did not achieve the targeted elimination [20]. Following SAT, tsetse control was sustained to date with low-cost tools (i.e. ITC and ITT) and at a low intensity, with a focus on the regional border with the Mole National Park to stem the reinvasion pressure. The pre-intervention situation is shown in plate 2005–2009, which includes the baseline data collected in 2008 and 2009 [12], while the plates from 2010–2014 onwards show the entomological monitoring carried out during and after the deployment of SAT. Notwithstanding sources of bias such as differences in the locations, intensity and seasonal timing of the surveys, Fig. 3 and Table 2 suggest a sizeable and sustained reduction in tsetse fly densities of approximately 78%.

**Table 2:** Intensity and results of entomological surveillance for tsetse flies in the Upper West Region

Period	Intensity of surveillance	Tsetse flies caught	
	[Trap days]	[No.]	[Flies/trap/day]
2005–2009	544	3561	6.55
2010–2014	627	2748	4.38
2015–2019	206	283	1.37
2020–2025	185	266	1.44

Periods: 2005–2009, 2010–2014, 2015–2019 and 2020–2025

Trapping device: biconical trap[43]

### African animal trypanosomosis

**Fig. 4:**
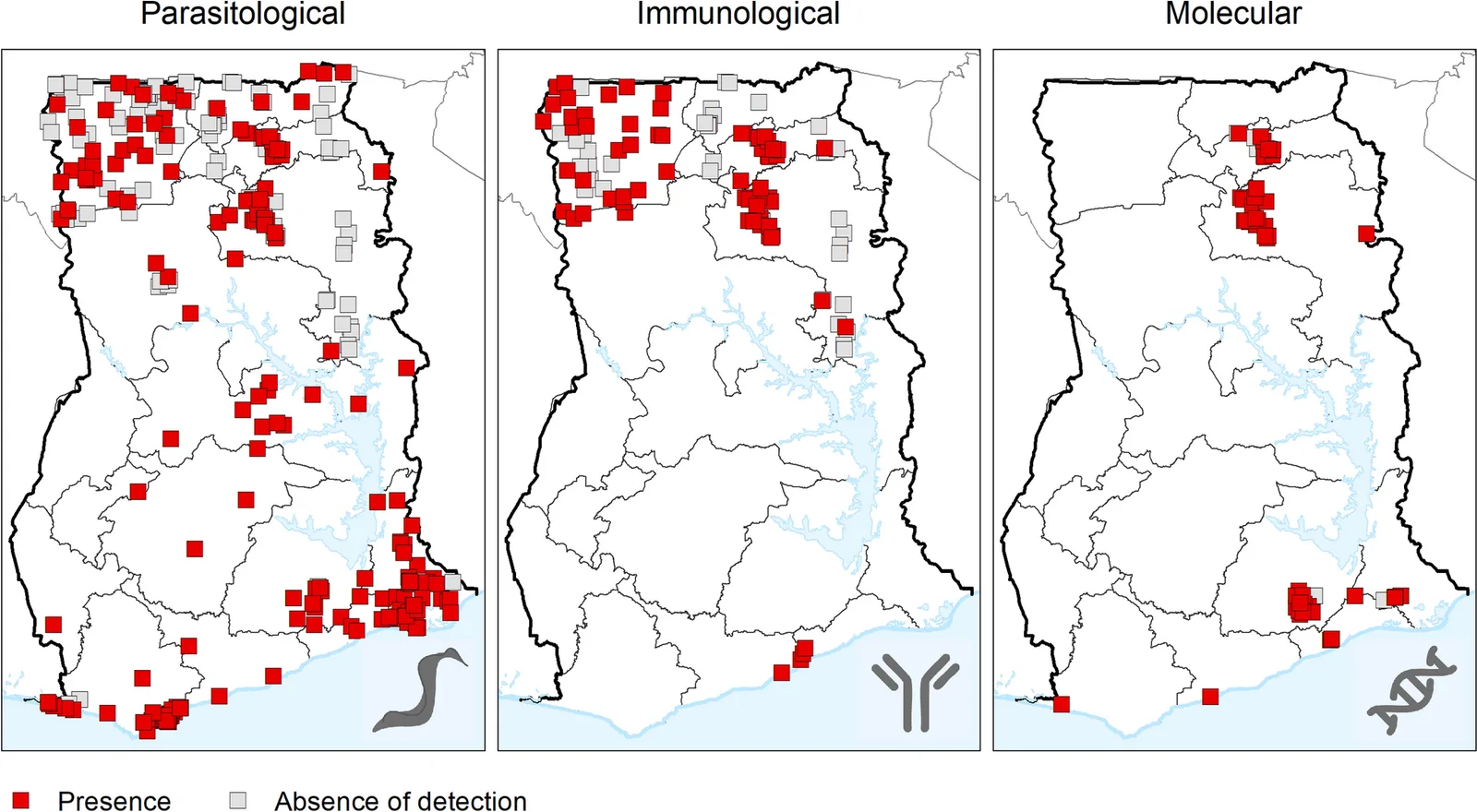
Presence (red squares) or absence of detection (grey squares) of African animal trypanosomosis in Ghana based on three categories of diagnostic techniques, i.e. parasitological, immunological and molecular. Data collection period: 1992–2025

Figure 4 summarizes the data of the AAT national atlas in Ghana by showing the presence or absence of detection of the disease, disaggregated for the categories of diagnostic techniques (i.e. parasitological, immunological and molecular). In this figure all livestock species are included.

Figure 4 shows that the traditional parasitological techniques are the most widely used. Blood smears are the main diagnostic tool used in routine disease surveillance, while buffy coat technique (BCT) is most frequently employed in epidemiological surveys. Immunological and molecular techniques remain the preserve of more targeted surveys in which a research component is included.

**Fig. 5:**
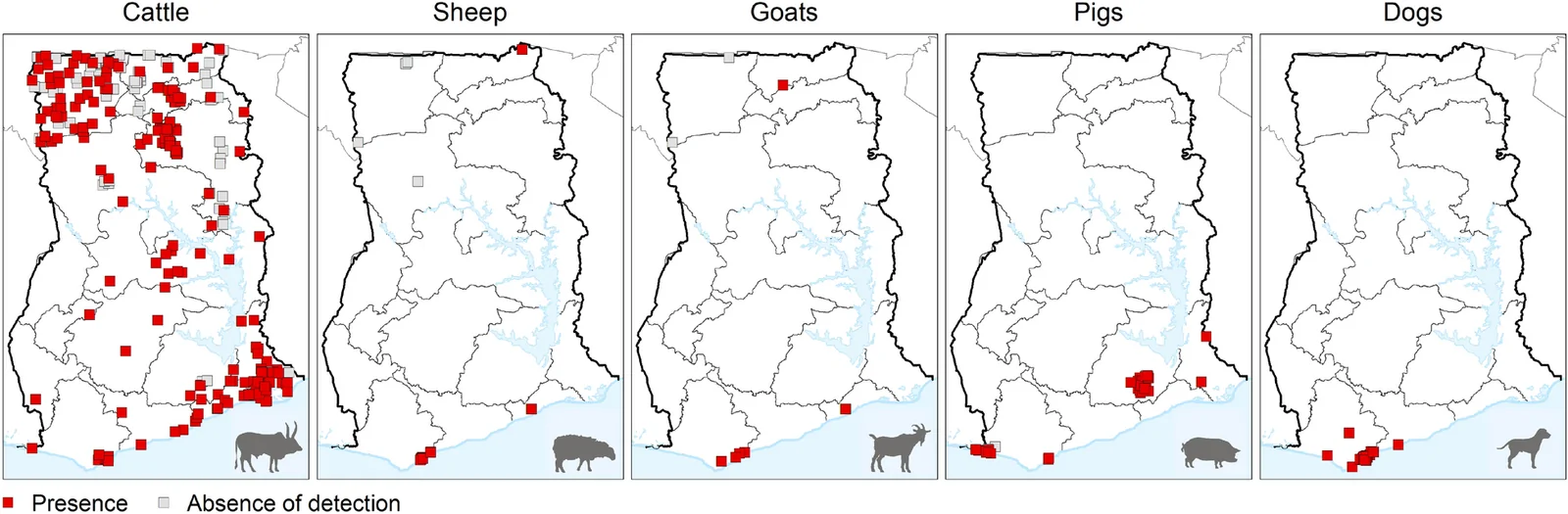
Presence (red squares) or absence of detection (grey squares) of African animal trypanosomosis in Ghana by animal species. Data collection period: 1992–2025

Figure 5 summarizes AAT data by animal species. Considering that cattle represent approximately 88% of the available data, and that data for other animal species are very sparse, from now on the presentation of the results of the AAT component of the atlas we focus on bovine trypanosomosis.

**Fig. 6:**
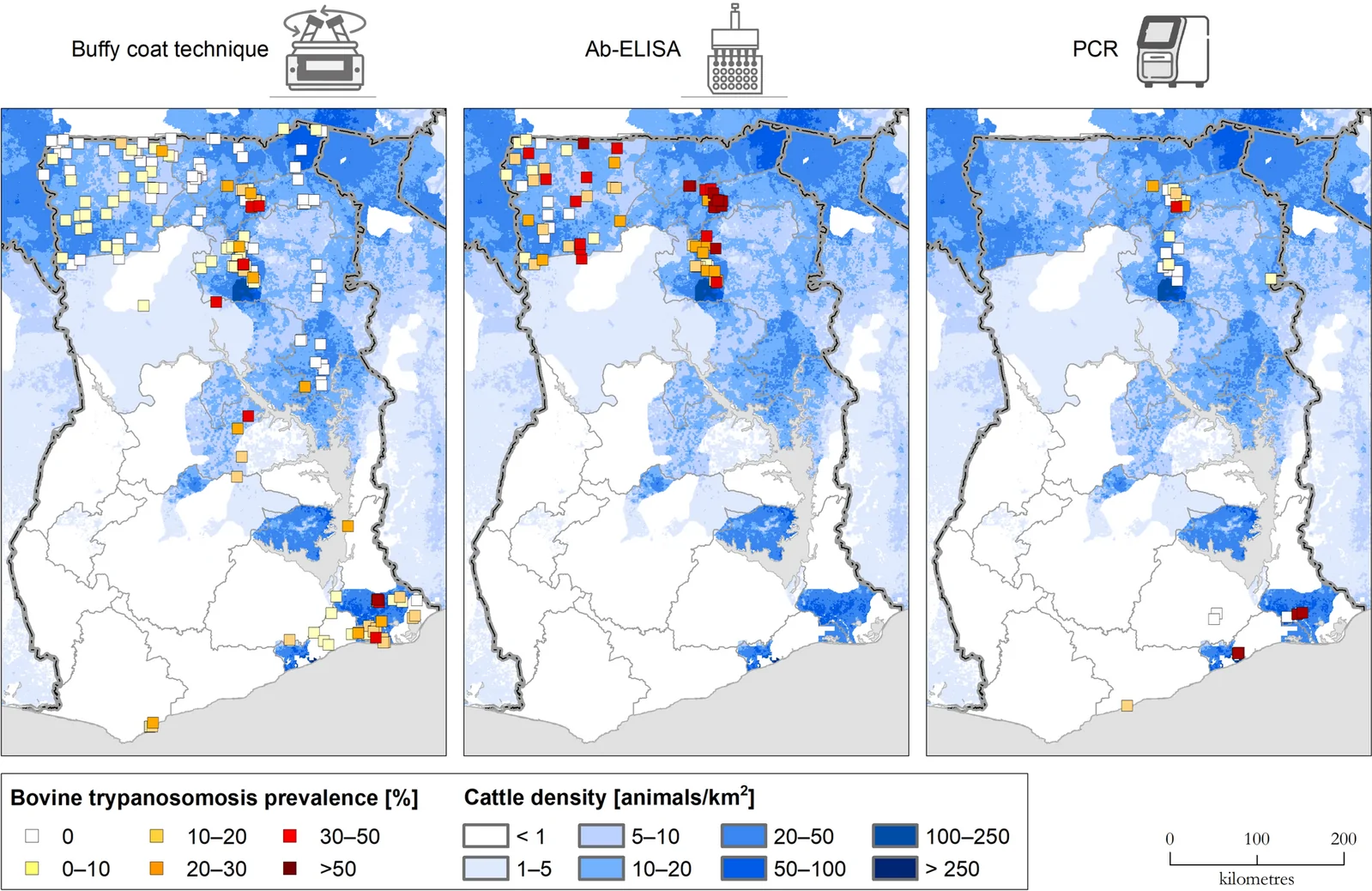
Prevalence of bovine trypanosomosis in Ghana based on different diagnostic techniques, i.e. buffy coat technique, Ab-ELISA and PCR. Type of animal sampling: random. Data collection period: 1992–2025

Figure 6 and Table 3 show the prevalence of bovine trypanosomosis for the three main diagnostic techniques, i.e. BCT, Ab-ELISA and polymerase chain reaction (PCR), which account for 75% of the surveys based on a random selection of animals. In Fig. 6, the density of bovines per km^2^, as provided by the FAO Gridded Livestock of the World [49], is used as a background. We note that surveys were mostly focused on the northern part of the country, as well as in the Volta and Greater Accra regions, which are areas with relatively higher cattle populations. An overall prevalence of 7.7% was detected with BCT, while for both Ab-ELISA and PCR the measured prevalence was almost 3 times higher (i.e. 22% and 17% percent, respectively).

**Table 3:** Prevalence of bovine trypanosomosis in Ghana based on different diagnostic techniques, i.e. buffy coat technique, Ab-ELISA and PCR

Region^1^	BCT			Ab-ELISA			PCR		
	Animals tested	Positive animals		Animals tested	Positive animals		Animals tested	Positive animals	
	[No.]	[No.]	[%]	[No.]	[No.]	[%]	[No.]	[No.]	[%]
Bono East	548	59	10.8	0			0		
Central	0			0			55	7	12.7
Eastern	890	63	7.1	0			20	0	0.0
Greater Accra	2320	248	10.7	0			254	126	49.6
North East	745	103	13.8	505	261	51.7	505	66	13.1
Northern	2441	194	7.9	972	116	11.9	930	48	5.2
Savannah	110	20	18.2	0			0		
Upper East	460	2	0.4	0			0		
Upper West	2682	52	1.9	1800	343	19.1	0		
Volta	494	75	15.2	0			185	85	45.9
Western	76	9	11.8	0			0		
Total	10,766	825	7.7	3,277	720	22.0	1,949	332	17.0

Type of animal sampling: random

Data collection period: 1992–2025

^1^No data available for Ahafo, Ashanti, Bono, Oti and Western North regions

**Fig. 7:**
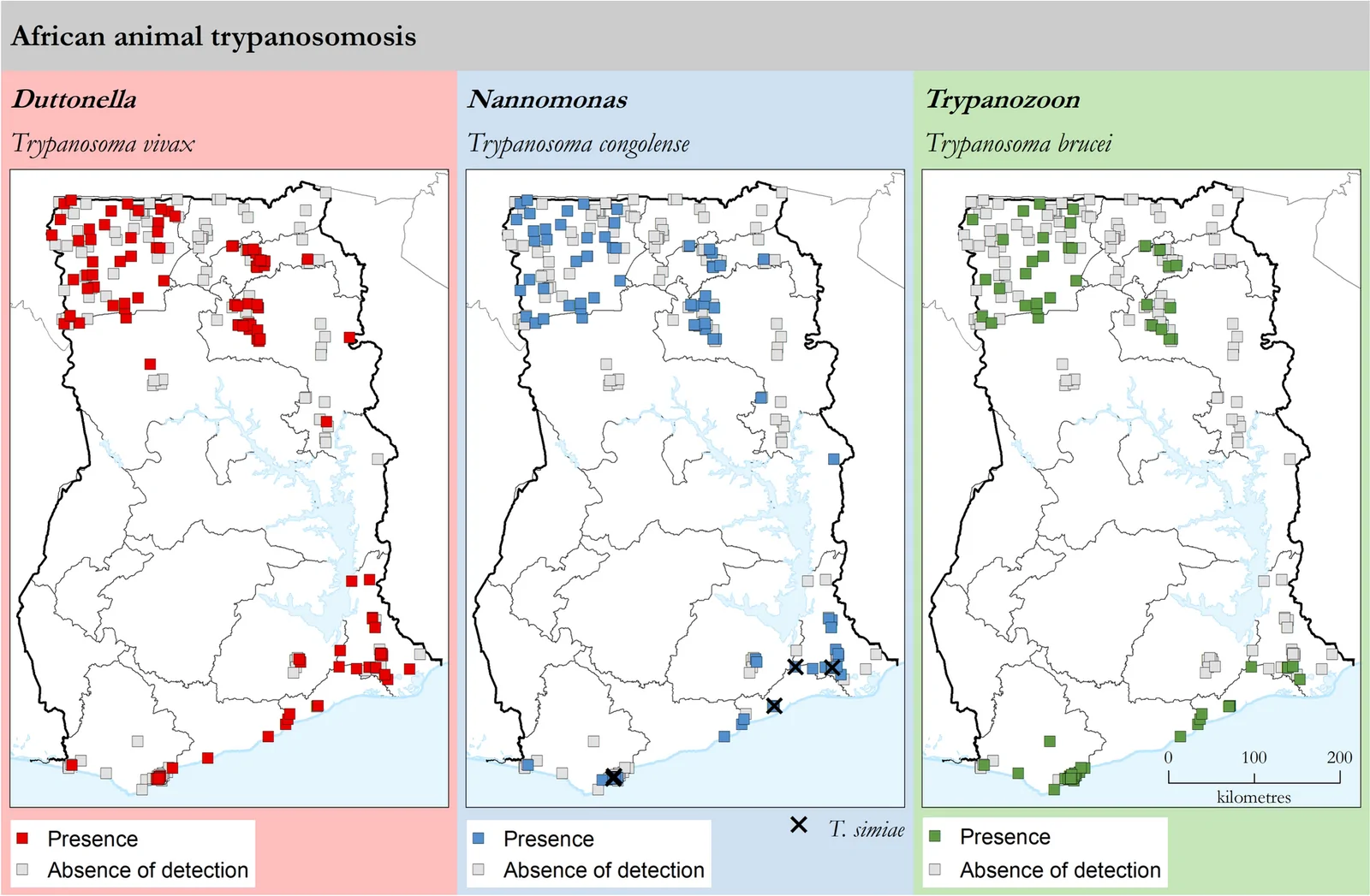
Presence (coloured squares) or absence (surveyed but not detected, grey squares) of *T. vivax*, *T. congolense* and *T. brucei* in Ghana. Presence of *T. simiae* is shown as black crosses in the plate for *T. congolense*. Data collection period: 1992–2025

Figure 7 summarizes the AAT atlas data at the trypanosome species level, thus including four species: *Trypanosoma vivax*, *T. congolense*—combined here in the same map with *T. simiae* – and *T. brucei*. Figure 7 shows that, with the exception of *T. simiae*, the other 3 trypanosomes are widespread at the country level.

### Database completeness

For tsetse database, completeness above 90% was achieved for most of the columns. In particular, data source file, geographical coordinates, administrative unit 1 (region), number of traps, trap/site level, coordinates source, and presence/absence were 100% complete, while a 98% level was reached for location name, survey period, trap type, and trap attractant; high levels of completeness (i.e. 97%) were also found for tsetse species (along with their respective apparent density), trapping duration, and tsetse intervention activity, while administrative unit 2 (district) was 89% complete. Elevation was only 33% complete (however, elevation is not considered a key variable for the atlas database because it can be easily extracted in GIS from public domain Digital Elevation Models).

For AAT database, 100% completeness was achieved for data source file, geographical coordinates, location name, administrative unit 1 (region), animal species, and diagnostic test used (i.e. the three levels of categorization). AAT presence and number of infections were almost complete as well (100% and above 97%, respectively), although when we look at their occurrence at taxa level, the percentage is lower (e.g. for *T. vivax:* 76% and 73%, respectively, 75% and 73% for *T. congolense*), and the same applies to the respective prevalence columns. Almost 99% completeness was reached for sample size, and other elements such as animal age and sex (i.e. 98%), animal breed (97%), and the key element that is the sampling method (almost 97%). Husbandry system was 93% complete, while administrative unit 2 (district), tsetse interventions, and chemotherapy were filled at 92%, 89%, and 86%, respectively. Finally, PCV completeness was only 33%.

## Discussion

Reviewing and mapping data collected over a period of 34 years enabled to obtain a meaningful epidemiological picture of the tsetse and AAT in Ghana.

As regards tsetse distribution, the data assembled for this atlas confirmed the presence of tsetse in all 12 study regions, and historical data suggest that the flies are also present in the 4 remaining regions [21, 50]. However, this does not necessarily mean that all areas in the country are infested. For example, no fly was caught in 17 of the 56 surveyed districts, even though it should be considered that the intensity of surveillance in most of these districts was so low that the presence of tsetse cannot be ruled out. More in general, the geographical gaps in the entomological record are still large, with no data available for over 75% of the districts in the country.

As regards the different tsetse species, only 3 out of the 10 historically reported were detected: i.e. *G. tachinoides*, *G. palpalis*, and *G. morsitans*. *G. tachinoides* was confirmed to be the dominant species in the northern part of the country, but it was detected also in the centre and the south, and closer to the Atlantic coast than historically reported [19, 50]. This apparent southwards shift of the distributional limit of *G. tachinoides* seems to be in line with similar observations in other countries in the region such as Togo [51] and Nigeria [52, 53]. *G. palpalis* appears to be confirmed as the species with the broadest distribution, with higher apparent densities in the south and centre, but with a presence also in the north, albeit at lower densities. It should be noted here that two subspecies of *G. palpalis* are present in Ghana (*G. palpalis gambiensis* in the North and *G. palpalis palpalis* in the South). However, subspecies identification is not normally carried out by the TTCU, so results are recorded in the atlas at the species level. In the future, it could be interesting to explore the current distribution limit between the two subspecies of *G. palpalis* in Ghana, following the example of countries such as Côte d’Ivoire [38]. Of the three species detected*, G. morsitans* is the one with the most fragmented distribution and overall lower densities. It is mainly, albeit not exclusively, limited to protected areas, where it can find the wildlife hosts it normally relies on for feeding [54]. The progressive shrinking of the distribution of *G. morsitans* and its retrenchment in protected areas has been documented already in other countries in the region such as Burkina Faso, Mali and Nigeria [4, 33, 35, 55]. *G. morsitans submorsitans* is the only subspecies of *G. morsitans* present in western Africa, and therefore all *G. morsitans* captured in Ghana can be assumed to belong to *G. morsitans submorsitans*.

Of the seven tsetse species not detected during our study period, but historically reported, 4 belong to the forest/*fusca* group, 2 to the riverine/*palpalis* group, and 1 to the savannah/*morsitans* group. For most of them the lack of detection is probably explained by the very limited or non-existent surveillance in their historical distribution zones, and especially in the more densely forested areas in the southwestern part of the country [21]. However, for many of them a second factor affecting the lack of detection is likely to be an actual shrinking of their distribution due to land cover and land use change, and most notably deforestation [4, 56].

As regards AAT, despite the gaps in the present atlas, the disease is confirmed to be widespread across the country. The prevalence of bovine trypanosomosis in the studied areas, as measured by the most commonly used diagnostic method (BCT), is 7.7%. Comparisons of cumulative data from different national atlases should be made with care, because of the many sources of bias such as differences in time frame, geographical coverage and overall surveillance effort as measured by the number of animals tested. That said, the 7.7% BCT prevalence reported here for Ghana is a relatively high value if compared with the prevalence measured with the same method in national atlases of other countries such as Burkina Faso (6%, [35]), Ethiopia (4.8%, [32]), Zambia (4.4%, [39]), Mali (3.5%, [33]) and Kenya (1.9%, [34]).

Despite the usefulness of this first edition of the national atlas in Ghana, the tool is still affected by limitations. A notable one is the relatively weak overall intensity of entomological surveillance, which with approximately 5000 trap days constitutes a much small dataset compared to countries like Kenya, Ethiopia or Zimbabwe, where 15,000, 45,000 and 1 million trap days were compiled, respectively [32, 34, 40]. In relation to AAT, a more general limitation of all atlases including the present one is that the aggregated results cannot be considered as unbiased assessments of disease prevalence at the national level; they represent an amalgamation of the surveys carried out in the country, with a range of temporal and geographical biases. These biases must be fully considered for a correct interpretation and usage of the data. A similar limitation also affects the tsetse component of the atlas, so that, for example, the relative abundance of the different tsetse species in the atlas cannot be considered as an unbiased estimation of the species abundance at the country level.

## Conclusions

The atlas of tsetse flies and AAT in Ghana can be considered as a very successful atlas initiative at the national level. Its most notable feature may be that, despite material external support to its development came to an end in 2018 [27], the TTCU sustained its development and regular update to this day, with only limited FAO guidance and backstopping.

Another key feature of this atlas is that it places Ghana in a good position to advance along the AAT PCP. In particular, the sustained reduction of tsetse densities in the Upper West region for more than a decade after the conclusion of the PATTEC project (Fig. 3), is an indicator of possible sustained AAT reduction. This notion seems to be corroborated by the interesting albeit limited AAT data collected in the region between 2023 and 2025, during which period over 1100 animals were tested, some with blood smears, some with BCT and some with rapid diagnostic tests [57], and no AAT case was detected. These data suggest that the Upper West region of Ghana could be considered at ‘advanced stage 2’ of the PCP (i.e. ‘sustainable and economically profitable AAT reduction achieved’ [1]), although, to confirm this, more data on the current prevalence of AAT in the region are needed for a robust comparison with the 2008–2009 baseline data [12]; and, ideally, economic data on the profitability of the achieved AAT reduction would be needed.

Sustained progress in AAT reduction in one major region, combined with the progressive expansion of surveillance and control activities in a few other regions in the northern part of the country, which took place in the past few years, might be considered sufficient to place Ghana at stage 2 of the PCP at the national level, according to the recently published FAO guidelines [1]. However, this can only be considered as a preliminary or provisional assessment; for full compliance with the requirements for stage 2, Ghana would need to develop a national strategy against AAT in line with the PCP, and the related control plan. Furthermore, more robust data on the sustained reduction in disease burden would be needed in the main intervention area (i.e. the Upper West region).

Finally, the adoption by Ghana of an open data policy for its atlas is a very welcome development. It follows similar recent decisions by Zambia [39] and Côte d’Ivoire [38], and it embraces the example and general recommendation set by FAO [58]. This policy will vastly broaden the range of applications of the atlas data and it will also enhance future updates of the continental atlas [4]. In particular, open data will enable more detailed analyses of the atlas data by scientists and researchers, thereby enhancing the scientific relevance of the initiative.

Notwithstanding these achievements, important surveillance and spatial data gaps remain in the atlas, which underscore the need for continued surveillance, data updating, and targeted field investigations to improve future national assessments.

## Supplementary Information

Additional file 1: Database. The database of the national atlas of tsetse flies and African animal trypanosomosis in Ghana.

## Data Availability

All data presented in this paper are available as Supplementary Information.
